# Synchrotron X-ray photoelectron spectroscopy study of sodium adsorption on vertically arranged MoS_2_ layers coated with pyrolytic carbon

**DOI:** 10.3762/bjnano.16.64

**Published:** 2025-06-10

**Authors:** Alexander V Okotrub, Anastasiya D Fedorenko, Anna A Makarova, Veronica S, Sulyaeva, Yuliya V Fedoseeva, Lyubov G Bulusheva

**Affiliations:** 1 Nikolaev Institute of Inorganic Chemistry, SB RAS, 630090 Novosibirsk, Russia; 2 Physical Chemistry, Institute of Chemistry and Biochemistry, Free University of Berlin, 14195 Berlin, Germany; 3 Helmholtz-Zentrum Berlin für Materialien und Energie, 14109 Berlin, Germany

**Keywords:** graphitic coating, molybdenum disulfide film, Na adsorption/desorption, sodium vapor, XPS

## Abstract

Hybrid materials consisting of molybdenum disulfide (MoS_2_) and graphitic-like carbon have great potential for practical application as anodes in high-performance sodium-ion batteries. In this work, to reveal the effect of carbon coating on the interaction of sodium with the MoS_2_ layers located vertically relative to the substrate, model experiments were carried out using synchrotron-radiation-induced X-ray photoelectron spectroscopy (XPS). Sodium vapor obtained by heating a sodium source was simultaneously deposited in vacuum on the surfaces of MoS_2_, pyrolytic carbon, and a hybrid sample obtained by transferring a pyrolytic carbon film onto the MoS_2_ film. According to XPS data, sodium easily penetrates into the space between the vertical layers of the uncoated film, and its interaction with MoS_2_ leads to the transformation of the original hexagonal structure into a distorted tetragonal one. Under the experimental conditions, sodium is unable to diffuse through the carbon film consisting of horizontally oriented graphene domains and is almost completely removed by annealing the sample at 773 K in ultrahigh vacuum. The presence of the underlying MoS_2_ film facilitates the diffusion of sodium through the graphitic coating, but not all of the deposited sodium reaches MoS_2_. As a result, the sodium-induced rearrangement of the carbon-coated MoS_2_ is less than that of the free MoS_2_ film, and annealing of the sodiated sample restores its structure. The obtained results demonstrate the important role of the graphitic coating in the development of viable MoS_2_-based electrodes for energy storage systems.

## Introduction

Sodium-ion batteries (SIBs) attract increasing interest as a low-cost alternative to lithium-ion batteries due to the abundance and wide availability of sodium. Research in this field is currently focused on developing new electrode materials to increase the capacity and cycle life of SIBs. Molybdenum disulfide (MoS_2_) has a layered structure and a high theoretical capacity of 669 mAh·g^−1^, so it is considered as a promising anode material for SIBs [1–2]. The large sodium ion can diffuse with a low energy barrier between the S−Mo−S layers due to the interlayer spacing of 0.62 nm and weak van der Waals interactions between them. At a sodium ion intercalation potential of about 1.4 V vs Na/Na^+^, the thermodynamically preferred 2H-MoS_2_ phase transforms into the metastable 1T-MoS_2_ phase [3–4]. With further increase in the intercalated sodium concentration (according to calculations above 1.75 Na per unit MoS_2_), the intercalate decomposes into amorphous Na_2_S and Mo; this reaction occurs at potentials below 0.8 V vs Na/Na^+^ [3]. The reaction products cannot be converted back to MoS_2_ due to the strong Na–S bonding [5]. The irreversible conversion reaction resulting in low electrical conductivity and huge volume expansion of the anode material limits the application of MoS_2_ anodes in high-energy SIBs. Thus, the main issues that need to be addressed for SIBs with MoS_2_ anodes are long-term stability and high rate performance.

Conducting graphitic-like carbon additives have been proposed as an effective way to solve the problem of electrical conductivity and stability of MoS_2_ anodes [6]. To date, several hybrid MoS_2_–carbon anode materials have been developed, which have demonstrated excellent cycling stability and rate performance in SIBs, as well as high reversible specific capacity [7–16]. Moreover, it has been reported that the electrochemical reaction of MoS_2_ with sodium ions could be reversible in the presence of graphitic components [17]. Wang et al. showed that in an anode material in which graphitic layers were sandwiched between MoS_2_ layers, the MoS_2_ component was not converted to Mo and Na_2_S even at a high degree of sodiation [18]. An ex situ study of a fully sodiated anode composed of MoS_2_ nanosheets coupled with few-layered graphene revealed a partial transformation of 2H-MoS_2_ into a distorted tetragonal structure without significant formation of Mo and Na_2_S [19]. The carbon coating improved the electrical contact between the MoS_2_ agglomerates, while the sandwich-like structure of MoS_2_-graphene facilitated the diffusion of sodium ions [20–21].

There are various possibilities to improve the properties of hybrid anodes based on MoS_2_. The diffusion of sodium ions in layered MoS_2_ is highly anisotropic. It is fast along the basal planes and is not possible through defect-free layers [3]. Thus, when the interlayer channels coincide with the Na^+^ movement paths, the diffusion distance is shortened, resulting in fast intercalation reaction kinetics. Flower-like MoS_2_–carbon hybrids have demonstrated superior alkali metal storage capability and high rate performance due to the fast Na^+^ diffusion in radially orientated ultrathin MoS_2_ and graphene layers; the latter component ensured high electron transfer and structural stability of the material [22–24].

The orientation of the carbon component in the hybrid can also affect the electrochemical processes. For example, interlayer-expanded MoS_2_ nanosheets vertically anchored on graphene film and carbon fibers showed a good rate performance in SIBs [25–26]. It was shown that carbon coating on MoS_2_ particles prevents their aggregation, increases conductivity and reduces structural expansion during electrochemical cycling [21,27]. Hybrid materials consisting of vertically oriented MoS_2_ layers and graphitic carbon coating with horizontal layer orientation remain poorly understood with respect to sodium-ion storage behavior. Comprehensive studies in this direction are needed.

In this work, a thin MoS_2_ film with vertically aligned layers was coated by a thin film of pyrolytic carbon (PyC) with predominantly horizontal orientation of graphitic domains. The resulting hybrid and individual films of MoS_2_ and PyC were placed on the same sample holder to study the interaction with sodium vapor in the ultrahigh vacuum (UHV) chamber of the experimental station of the Russian–German beamline at the BESSY-II synchrotron radiation facility. Such model experiments make it possible to differentiate the diffusion rate of sodium in the hybrid and to identify the possible synergistic effect of the components in their interaction with sodium. Previously reported similar model experiments on lithiation of graphene [28], MoS_2_ crystals [29–31], and MoS_2_–graphene heterostructures [32] demonstrated an advantage in studying the interaction of lithium with carbon and other elements of the materials. It should be noted that anode materials with alkali ions introduced during electrochemical reactions in SIBs are difficult to study because of the presence of electrolyte decomposition residues.

## Results and Discussion

**Figure 1:**
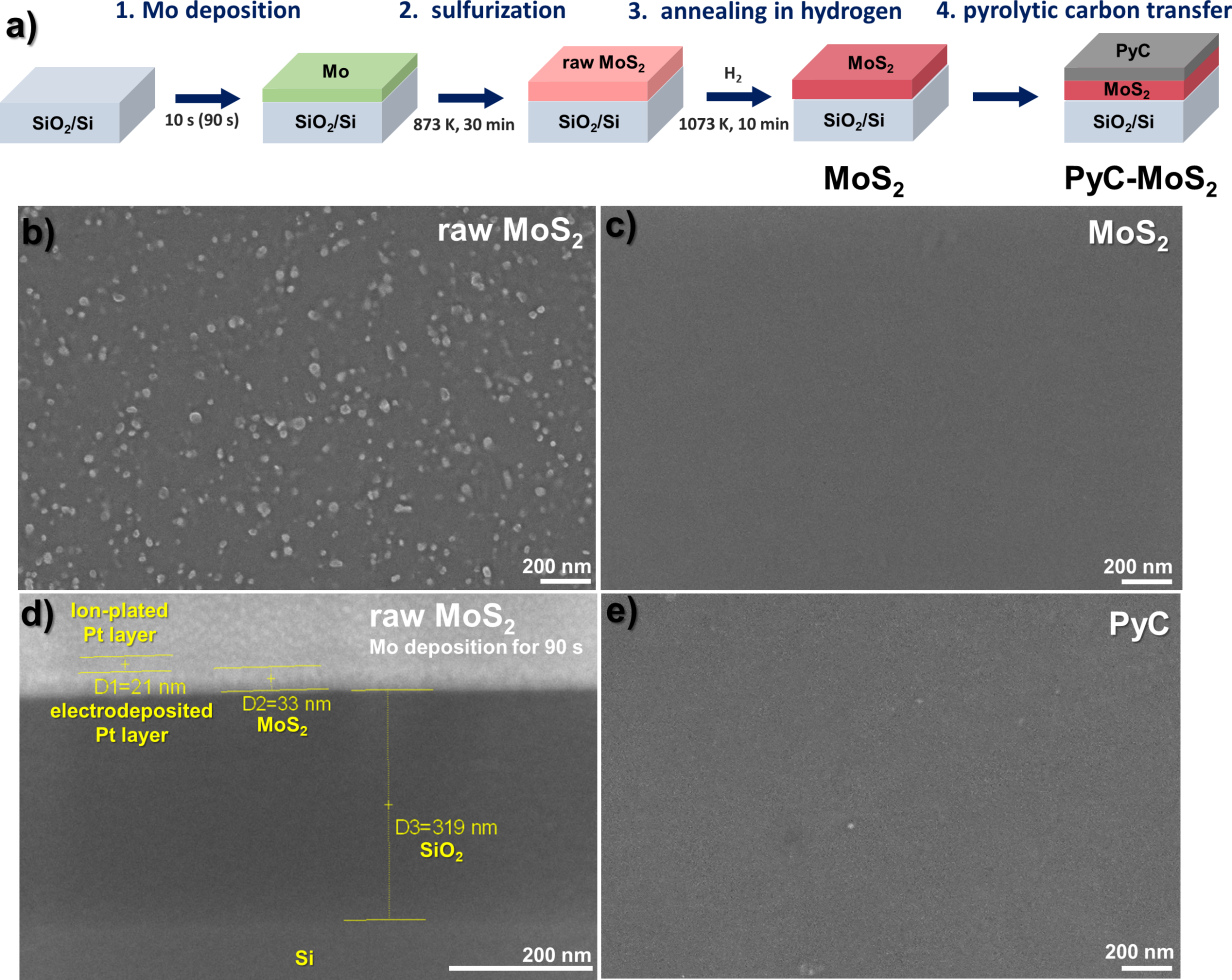
(a) Schematic diagram of the synthesis of MoS_2_ and PyC-MoS_2_ films. SEM images of the top view of (b) the raw MoS_2_ film obtained using a Mo layer sputtered for 10 s and (c) the film after heating in hydrogen. (d) Cross-sectional view of the MoS_2_ film obtained using a Mo layer sputtered for 90 s and (e) the top view of the PyC film on a SiO_2_/Si substrate.

Figure 1a shows the schematic diagram of the synthesis route of a hybrid film consisting of MoS_2_ coated with PyC. A molybdenum layer is deposited on a SiO_2_/Si substrate by magnetron sputtering for a short time of 10 s. This layer interacts with sulfur vapor at a temperature of 873 K for 30 min. Heating the raw film in a hydrogen atmosphere at 1073 K removes excess sulfur and other contaminations from the film surface. In the final step, a thin PyC film synthesized by chemical vapor deposition (CVD) technique is placed on the surface of the cleaned MoS_2_ film using the wet transfer method (see the Experimental section for details). The resulting hybrid, designated PyC-MoS_2_, together with a surface-cleaned MoS_2_/SiO_2_/Si sample and a PyC film transferred onto a SiO_2_/Si substrate, were used to comparatively study the ability to adsorb and accumulate evaporated sodium.

The scanning electron microscopy (SEM) images of the surface of raw MoS_2_ film, hydrogen-annealed film, and PyC film are compared in Figure 1b,c,e. The raw MoS_2_ film covers the entire area of the substrate and contains polysulfide nanoparticles on the surface (Figure 1b). These nanoparticles are absent on the surface of the MoS_2_ film annealed in a hydrogen atmosphere (Figure 1c). An attempt to measure the cross section of this film did not yield a contrast image because of the charging effect. Therefore, to estimate the thickness of the studied film, we used a thicker MoS_2_ film synthesized with a molybdenum layer sputtered for 90 s. Part of the film surface was covered with a protective Pt layer and a lamella was cut using a focused ion beam (FIB) system (see the Experimental section for details). Figure 1d shows the SEM image of the cross section of the lamella. The bright round spots on the film surface correspond to Pt nanoparticles, the presence of which is confirmed by energy-dispersive X-ray (EDX) spectroscopy (Supporting Information File 1, Figure S1). These nanoparticles have a uniform size and are densely distributed on the sample surface, in contrast to the polysulfide particles of different sizes formed during CVD synthesis (Figure 1b). The thickness of the MoS_2_ film estimated from the cross-sectional SEM image is about 33 nm (Figure 1d). Therefore, it can be estimated that the MoS_2_ film obtained using a molybdenum layer sputtered for 10 s has a thickness of no more than 4 nm. The SEM image of PyC transferred onto the SiO_2_/Si substrate also shows a uniform film surface (Figure 1e).

**Figure 2:**
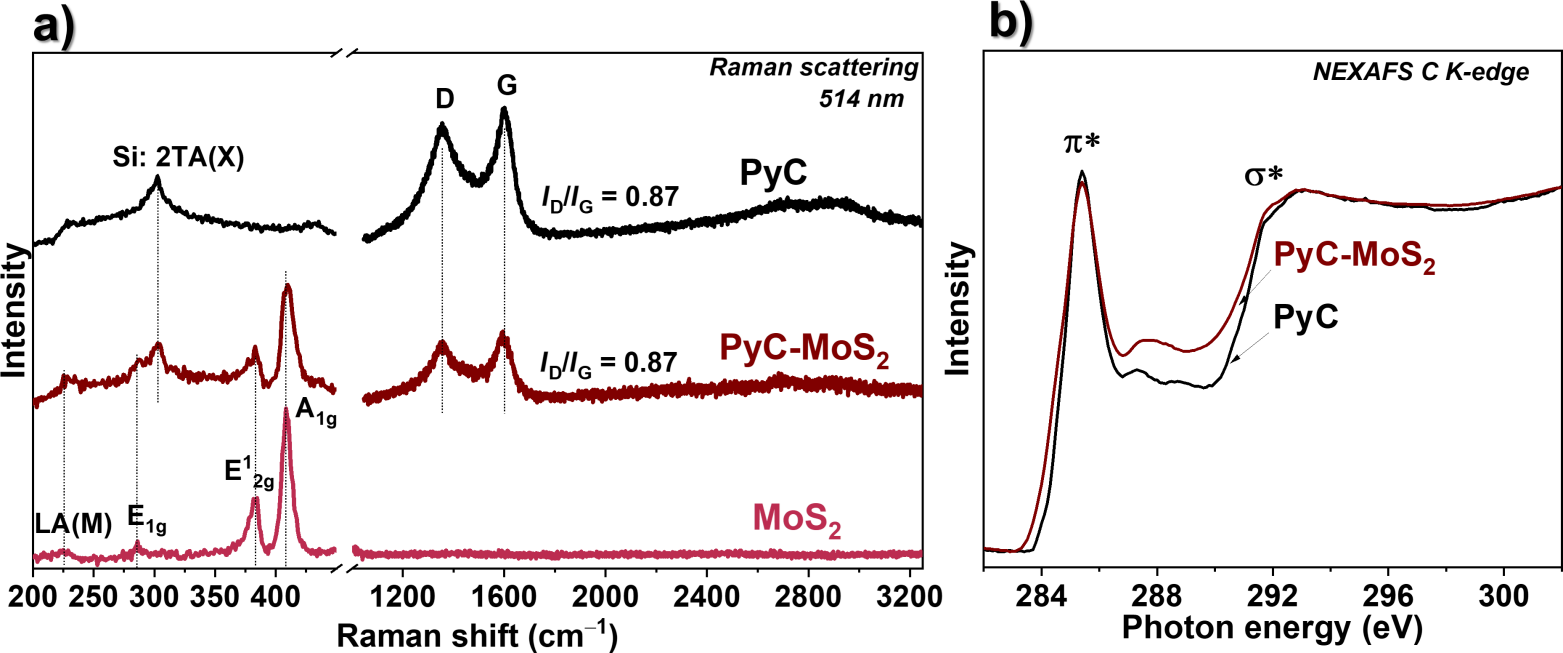
(a) Raman spectra of MoS_2_, PyC-MoS_2_, and PyC films. (b) NEXAFS C K-edge spectra of PyC and PyC-MoS_2_ films.

The Raman spectrum of the MoS_2_ film contains two strong peaks at 382.6 and 408.9 cm^−1^ (Figure 2a) corresponding to the E^1^_2g_ mode and the A_1g_ mode, respectively, of 2H-MoS_2_ [33]. The difference between the positions of these peaks is often used to determine the number of layers in MoS_2_ particles [34]. The distance between the peaks of ≈26.3 cm^−1^ for the MoS_2_ film is similar to that for bulk MoS_2_ [35]. Because of the small thickness of the MoS_2_ film, such a large number of the layers can be realized only when they are oriented vertically to the substrate surface. The weak peak at about 280 cm^−1^ observed in the Raman spectrum corresponds to the E_1g_ mode, which is forbidden when the laser beam is incident perpendicularly on the *c* axis of MoS_2_ [36]. The activation of this mode in our case confirms the vertical orientation of the MoS_2_ layers relative to the substrate surface [37]. The weak defect-induced mode LA(M) at 227 cm^−1^ and the asymmetric shape of the E^1^_2g_ and A_1g_ modes indicate the nanometer size of the MoS_2_ crystallites in the plane [38–39]. All the above modes are visible in the Raman spectrum of the PyC-MoS_2_ sample, so the coating with PyC film does not destroy the structure of the MoS_2_ film. The Raman spectra of PyC and PyC-MoS_2_ show a peak at 1600 cm^−1^ corresponding to the in-plane stretching of C=C bonds (G mode) and a peak at 1355 cm^−1^ caused by the disorder in the graphite lattice (D mode) [40]. The position of the G mode is higher than the position of the G peak at 1582 cm^–1^ for crystalline graphite and graphene [41], indicating the disorder in the layers and their functionalization. In fact, the intensity ratio of the D to G peaks (*I*_D_/*I*_G_) of 0.87 is relatively high. The weak second-order band between 2700 and 2900 cm^‒1^ is due to the three-dimensional ordering along the *c* axes of the graphitic film.

The NEXAFS spectra measured at the C K-edge of PyC and PyC-MoS_2_ films pre-annealed in UHV at 673 K for 10 min exhibit two main resonances located at 285.4 and 291.8 eV (Figure 2b), which are attributed to the electron transitions from the C 1s core levels to the π* and σ* C=C states in the graphitic structure, respectively [42]. The rather sharp shape of the π* resonance indicates the graphitic-like structure of the PyC film. Weak features appearing between the π* and σ* resonances suggest that the PyC film is slightly functionalized with oxygen- and/or hydrogen-containing groups. The spectrum of the PyC-MoS_2_ film almost repeats the shape of the spectrum of the PyC film. A slight decrease in the intensity of the π* resonance and an increase in the intensity in the regions before and after the π* resonance at 284–285 eV and 286–289 eV can be associated with the interaction between PyC and MoS_2_ components [43]. The shift of the C K-edge spectrum of the PyC-MoS_2_ film toward lower photon energies corresponds to the electron density transfer from the carbon component to MoS_2_, as shown by density functional theory (DFT) calculations for the MoS_2_/graphene heterostructure [44–45]. According to the DFT calculations, the changes observed in the PyC-MoS_2_ spectrum between the π* and σ* resonances may result from the interaction of the π electrons of carbon with the p orbitals of sulfur [44].

**Figure 3:**
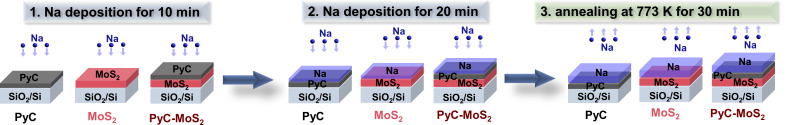
Schematic diagram of sequential processing of PyC, MoS_2_, and PyC-MoS_2_ films, comprising three steps: (1) sodium deposition for 10 min, (2) sodium deposition next 20 min, (3) vacuum annealing at 773 K for 30 min.

Figure 3 shows the sequence of a three-step sodiation/desodiation experiment performed with samples in the UHV chamber of the spectrometer. Sodium vapor was deposited simultaneously on three studied samples for 10 min. The second step included additional deposition of sodium for 20 min. The thickness of the sodium layer was measured using a quartz microbalance; it was 2.5 Å after the 10 min experiment and 7.6 Å after the 30 min experiment. In the third step, the samples with deposited sodium were annealed at 773 K for 30 min. The XPS spectra were measured before the three-step experiment and after each modification.

Survey XPS spectra of the samples revealed the presence of molybdenum, sulfur, carbon, sodium, and oxygen (Supporting Information File 1, Figure S2). The intense lines of silicon and oxygen detected in the spectrum of the PyC film are associated with the substrate. The absence of the Si 2p line in the spectra of MoS_2_ and PyC-MoS_2_ films indicates the formation of a continuous MoS_2_ film with a thickness of more than 3 nm [46]. The atomic concentrations of Mo and S in the MoS_2_ film are about 9 and 28 atom %, respectively, and decrease to about 2 and 7 atom % after coating the film with PyC.

**Figure 4:**
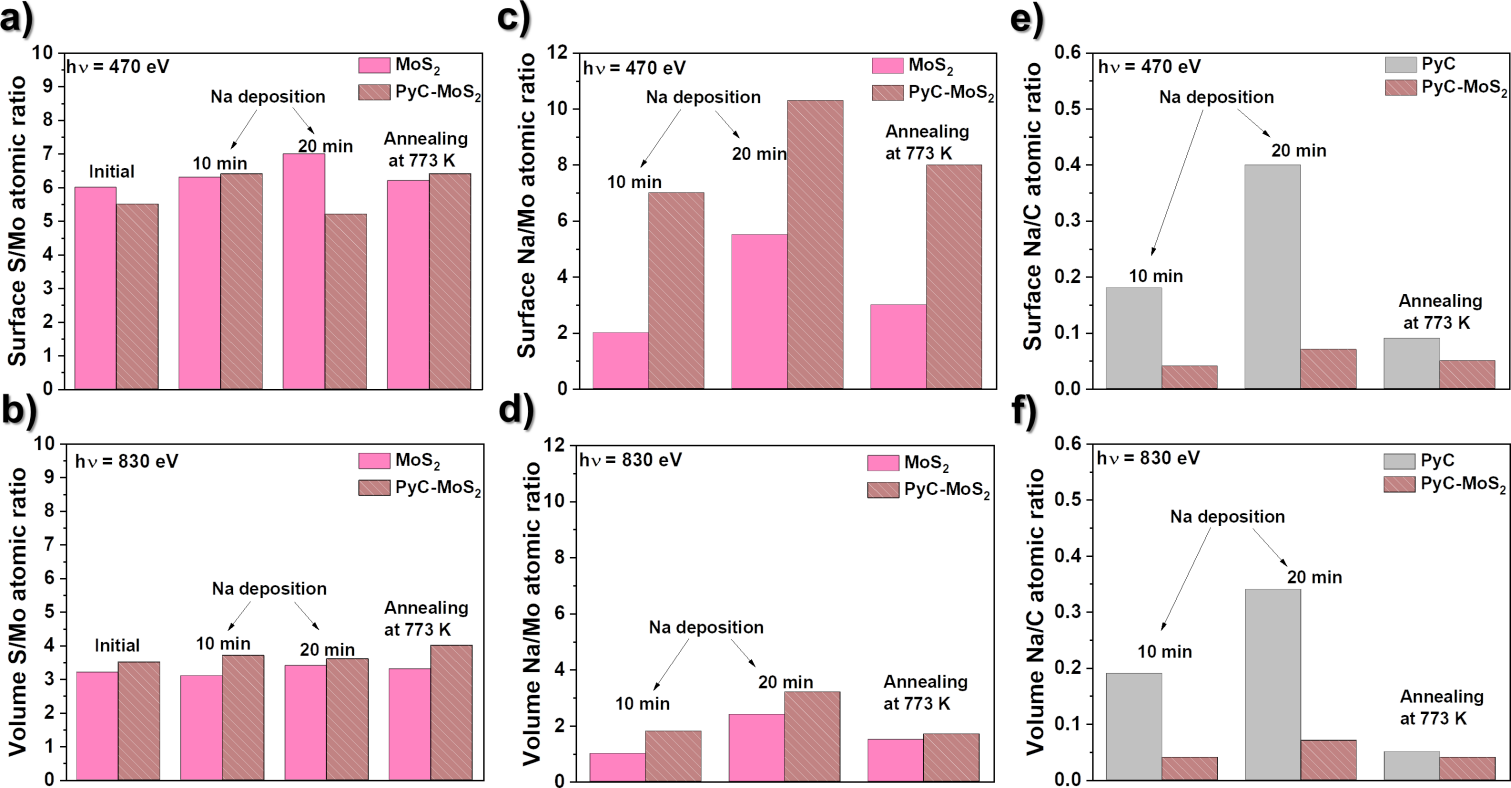
(a, b) XPS-derived atomic concentration ratios of sulfur to molybdenum (S/Mo), (c, d) sodium to molybdenum (Na/Mo), and (e, f) sodium to carbon (Na/C) directly on the surface (470 eV excitation, top line) and deeper from the surface (830 eV excitation, bottom line) of PyC, MoS_2_, and PyC-MoS_2_ films.

Figure 4 compares the S/Mo, Na/Mo, and Na/C ratios in the samples determined from the XPS survey spectra measured at excitation photon energies of 470 and 830 eV. The former energy provides a probing depth of about 1 nm and therefore allows for the determination of the surface composition of the films. At 830 eV, the probing depth is about 3 nm, which corresponds to almost the entire volume of the thin films under study. The S/Mo ratio in the MoS_2_ film is 6 on the surface and 3.1 in the bulk (Figure 4a,b). Excess sulfur in the MoS_2_ film is associated with the formation of polysulfide groups on the surface due to the synthesis conditions, including the increased content of sulfur vapor. An additional factor for the high S/Mo surface ratio is the vertical orientation of the MoS_2_ layers. The S/Mo values determined for the PyC-MoS_2_ sample and after deposition/removal of sodium deviate from the corresponding values for the initial MoS_2_ film by no more than 16% for the surface and 9% for the bulk. The deviations may be due to the fact that it is practically impossible to record spectra from the same place on the sample, which is repeatedly moved between the preparation and measurement chambers.

The Na/Mo ratio on the surface of the MoS_2_ film is 2.0 after Na deposition for 10 min, 5.5 after additional Na deposition for 20 min, and 3.0 after annealing (Figure 4c). The corresponding Na/Mo ratios in the bulk of the MoS_2_ film are 1.0, 2.4, and 1.5 (Figure 4d). The Na content in the bulk is approximately two times smaller than that on the surface because of the slower Na diffusion rate as compared to the deposition rate. However, the increase in the sodiation time leads to an increase in the sodium content not only on the surface but also in the interior of the film. After annealing, the Na/Mo ratio decreased both on the surface and in the bulk of the MoS_2_ film. The results show that sodium can easily penetrate into the film consisting of vertically aligned MoS_2_ layers and be partially released during annealing.

The concentration of Na in the PyC-MoS_2_ film determined from the XPS survey spectra measured at 830 eV is about 6 atom % after sodium vapor deposition for 10 min, and this value does not change after an additional deposition of 20 min (Supporting Information File 1, Figure S2). This suggests that in the hybrid film, Na was not trapped in the upper PyC layer, but penetrated deeper into MoS_2_. The similar Na/C ratios for the surface (Figure 4e) and bulk (Figure 4f) of the PyC film and the PyC-MoS_2_ film indicate that sodium is fairly uniformly distributed within the carbon component. Thus, the sodiation of the PyC-MoS_2_ film results in Na/C ratios of 0.05 and 0.07 after Na deposition for 10 min and additional 20 min, respectively. These values are about five times lower than those in sodiated PyC, indicating that sodium preferentially passes through the PyC film to be stored on the surface of MoS_2_ rather than within its volume. The Na/C ratio in the annealed sodiated PyC-MoS_2_ is similar to that of PyC. The PyC-MoS_2_ hybrid film exhibits high recovery because a significant portion of Na is removed from the film surface after annealing, similar to what occurs with the pure PyC film.

**Figure 5:**
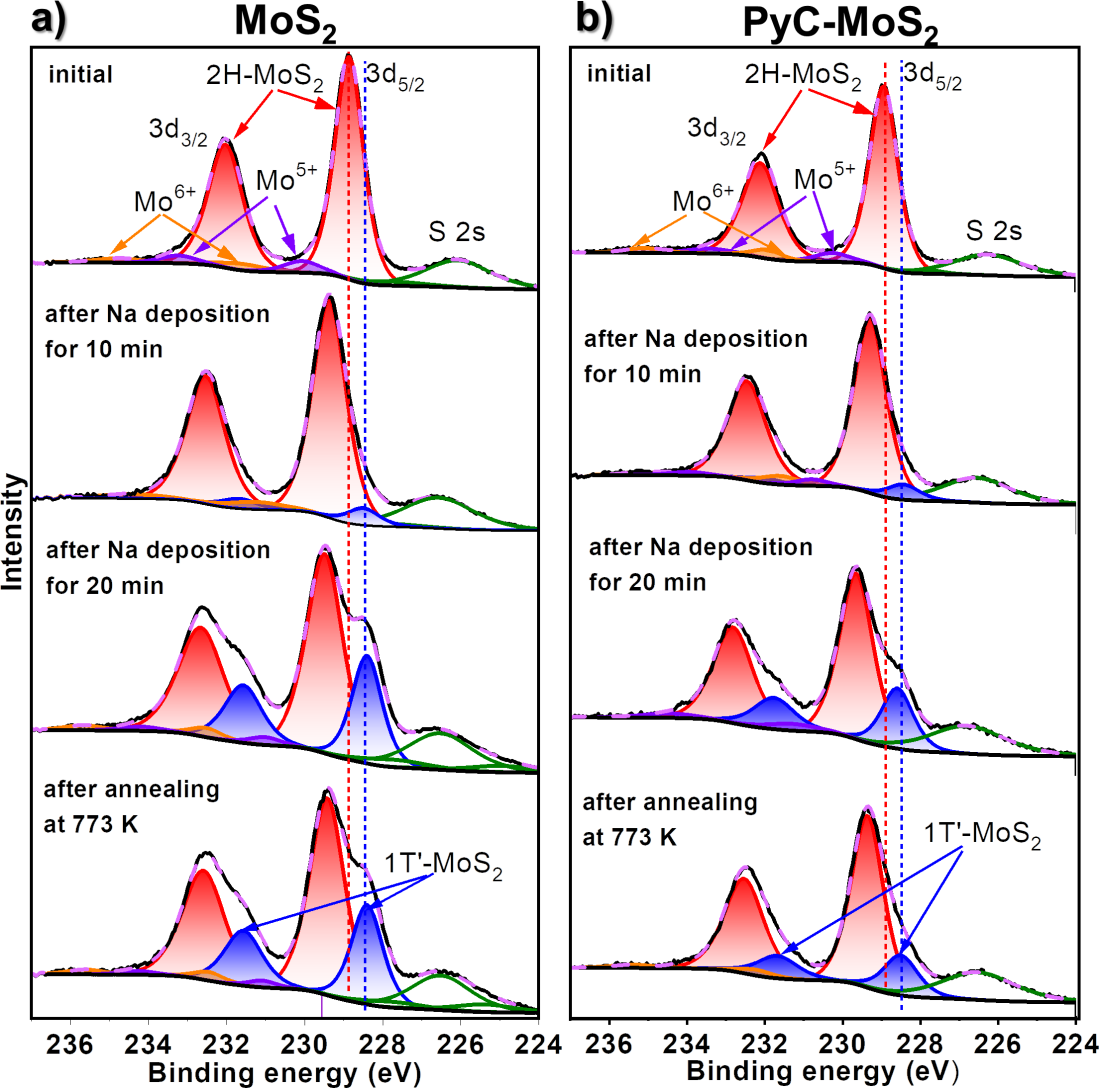
XPS Mo 3d spectra of (a) MoS_2_ and (b) PyC-MoS_2_ before and after sodium deposition for 10 min and additional 20 min and then after vacuum annealing at 773 K. The spectra were measured at 830 eV.

A comparison of the XPS Mo 3d spectra of MoS_2_ and PyC-MoS_2_ films is shown in Figure 5. The low-energy peak at 226.1–226.3 eV corresponds to the S 2s line. The Mo 3d spectra of the initial MoS_2_ and PyC-MoS_2_ consist of an intense spin–orbit doublet with the binding energy of the Mo 3d_5/2_ component of 228.9 eV (Figure 5a,b). This energy corresponds to the Mo^4+^ state in 2H-MoS_2_ [47]. In addition to the main peak, there are two weak doublets with Mo 3d_5/2_ binding energies of 230.1–230.3 eV and 231.6–231.9 eV, which belong to the oxidized forms of molybdenum in the Mo^5+^ and Mo^6+^ sates, respectively [48].

**Figure 6:**
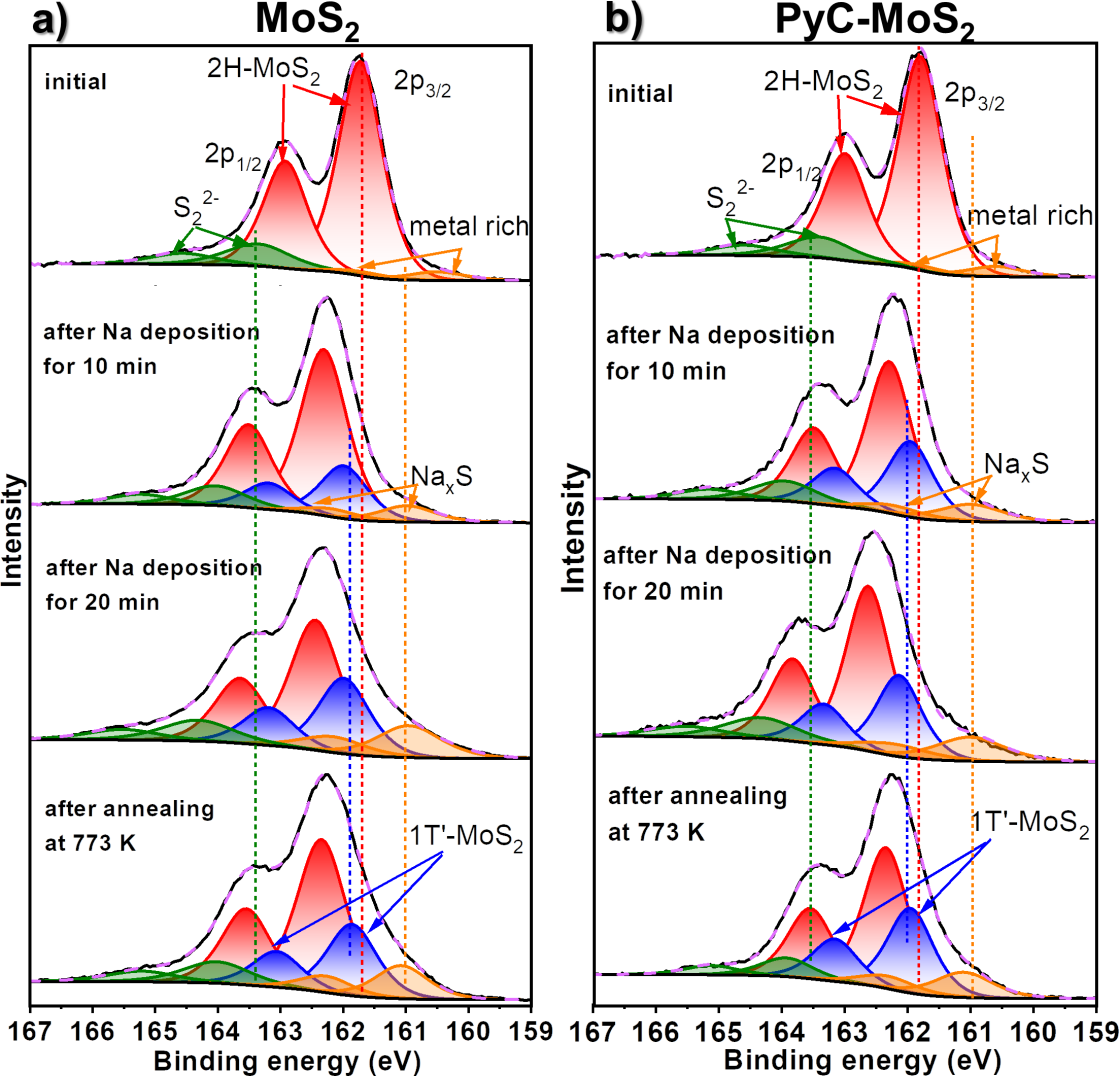
XPS S 2p spectra of (a) MoS_2_ and (b) PyC-MoS_2_ before and after sodium deposition for 10 min and additional 20 min and then after vacuum annealing at 773 K. The spectra were measured at 830 eV.

The XPS S 2p spectra of the initial MoS_2_ and PyC-MoS_2_ films exhibit an intense doublet with the S 2p_3/2_ component located at 161.7–161.8 eV (Figure 6a,b), corresponding to the S^2−^ sate [49]. In addition, the spectra contain two weak doublets, with the S 2p_3/2_ component at a binding energy of 163.4 eV, characteristic of S_2_^2−^ and polysulfide groups [50], and at 160.5 eV, associated with under-coordinated sulfur atoms formed at the MoS_2_ edges [51] as a result of preliminary sample annealing in H_2_ at 1073 K.

The Mo 3d (Figure 5) and S 2p spectra (Figure 6) of MoS_2_ and PyC-MoS_2_ films after sodium deposition exhibit additional low-energy doublets with the Mo 3d_5/2_ component at ≈228.5 eV and the S 2p_3/2_ component at ≈162.0 eV. These energies are characteristic for the distorted tetragonal 1T′-MoS_2_ [52]. Similar spectral changes were observed earlier after lithiation and sodiation of MoS_2_ and were associated with the transfer of electron density from alkali metals to MoS_2_, which led to the 2H‒1T′ transition [19,32,53–54]. Such structural transformations are accompanied by the formation of Mo‒Mo and Na‒S bonds and the weakening of S–Mo bonds [3]. In the spectra of sodiated films, the Mo 3d and S 2p doublets related to sodium-free 2H-MoS_2_ are retained, but their positions shift toward higher energies as compared to the spectra of the initial samples. The shift value of the Mo 3d and S 2p components of 2H-MoS_2_ increases with the deposited sodium concentration because of increased charge doping. The intensity of Mo 3d and S 2p components attributed to sodiated 1T′-MoS_2_ increases with sodium deposition time because more sulfur is bound to sodium. The areas of the 1T′-MoS_2_ doublet are similar in the Mo 3d spectra of MoS_2_ and PyC-MoS_2_ after sodium deposition for 10 min and constitute 7–8% of the total spectrum area (Figure 5). After additional sodium deposition for 20 min, the relative area of this doublet increases to 31% for the MoS_2_ film and to 25% for the PyC-MoS_2_ film. The smaller 1T′-MoS_2_ contribution in the latter case implies that the portion of sodium accumulated in the MoS_2_ structure of the carbon-containing PyC-MoS_2_ film is less than that in the bare MoS_2_ film. Annealing of the sodiated MoS_2_ and PyC-MoS_2_ in vacuum at 773 K leads to a decrease in the intensity of the 1T′-MoS_2_ doublet, which is more pronounced for the latter sample. Sodium is more easily released from the hybrid film because it is predominantly located on its surface and interacts more weakly with PyC than with MoS_2_.

**Figure 7:**
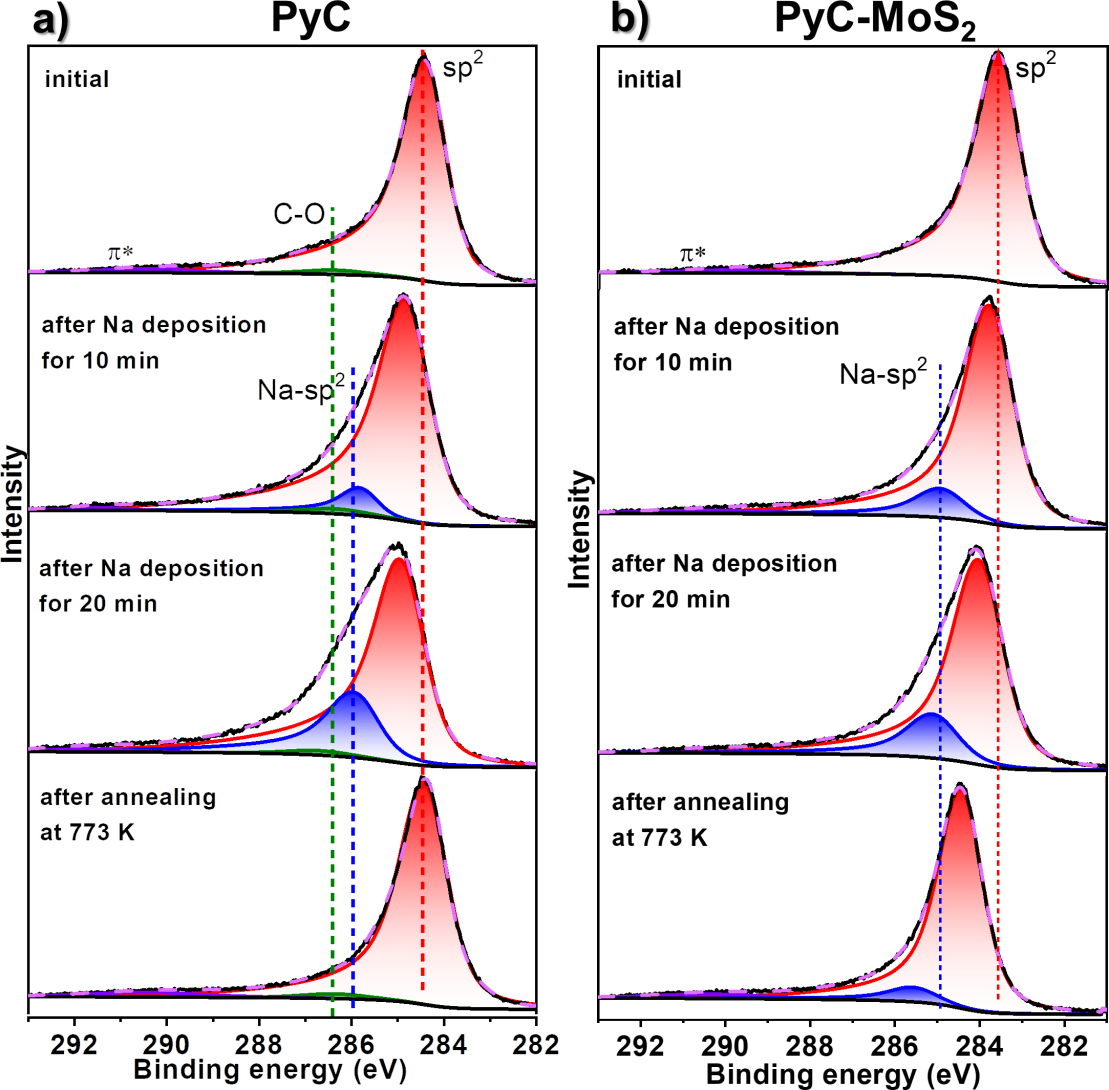
XPS C 1s spectra of (a) MoS_2_ and (b) PyC-MoS_2_ before and after sodium deposition for 10 min and additional 20 min and then after vacuum annealing at 773 K. The spectra were measured at 830 eV.

The analysis of XPS C 1s spectra of PyC and PyC-MoS_2_ films before and after sodium deposition followed by annealing is used to reveal the contribution of the PyC component to the interaction of PyC-MoS_2_ with sodium (Figure 7). The XPS C 1s spectrum of the PyC film shows an asymmetric peak at 284.4 eV (Figure 7a), which is typical for graphite-like carbon. In addition, there is a low-intensity component with a binding energy of 286.2 eV, corresponding to C–O bonds [55]. The C 1s spectrum of PyC-MoS_2_ has a similar shape (Figure 7b), indicating that the PyC films transferred onto the surface of the SiO_2_/Si substrate and the MoS_2_ film have the same structure.

After sodium deposition on the PyC film for 10 min and then 20 min, the sp^2^ peak shifts by 0.5 and 0.6 eV, respectively, towards higher binding energies. This shift is due to the charge transfer from sodium to the carbon layers. For PyC-MoS_2_, the shifts of the C 1s line caused by sodium deposition are smaller. A new high-energy component (Na-sp^2^) appearing at 286 and 285 eV in the spectra of sodiated PyC and PyC-MoS_2_ films, respectively, is due to carbon bonding with sodium. The intensity of this component is lower in the PyC-MoS_2_ spectrum measured after the total 30 min sodiation process. This is due to the lower charge transfer from sodium to the PyC component in the hybrid film as compared to the free PyC film, caused by its diffusion into the MoS_2_ component. According to the XPS data, sodium is redistributed between the components of PyC-MoS_2_.

After annealing, most of the sodium was removed from the PyC film, since the C 1s spectrum measured after this treatment completely returned to the spectrum of initial PyC (Figure 7a). In contrast to the PyC film, a significant portion of sodium remained in the PyC-MoS_2_ film after annealing. The shift of the sp^2^ component by 0.1 eV and the presence of a weak Na-sp^2^ component in the spectrum (Figure 7b) confirm that residual sodium interacts with carbon component.

The XPS Na 2s spectra of sodiated samples before and after annealing are presented as a single symmetric peak located at a binding energy of ≈65 eV for the PyC film and at ≈64 eV for the MoS_2_ film (Supporting Information File 1, Figure S3). The Na 2s spectrum of PyC-MoS_2_ film exhibits one peak at an intermediate position of 64.4 eV, confirming that Na binds with both PyC and MoS_2_ components.

**Figure 8:**
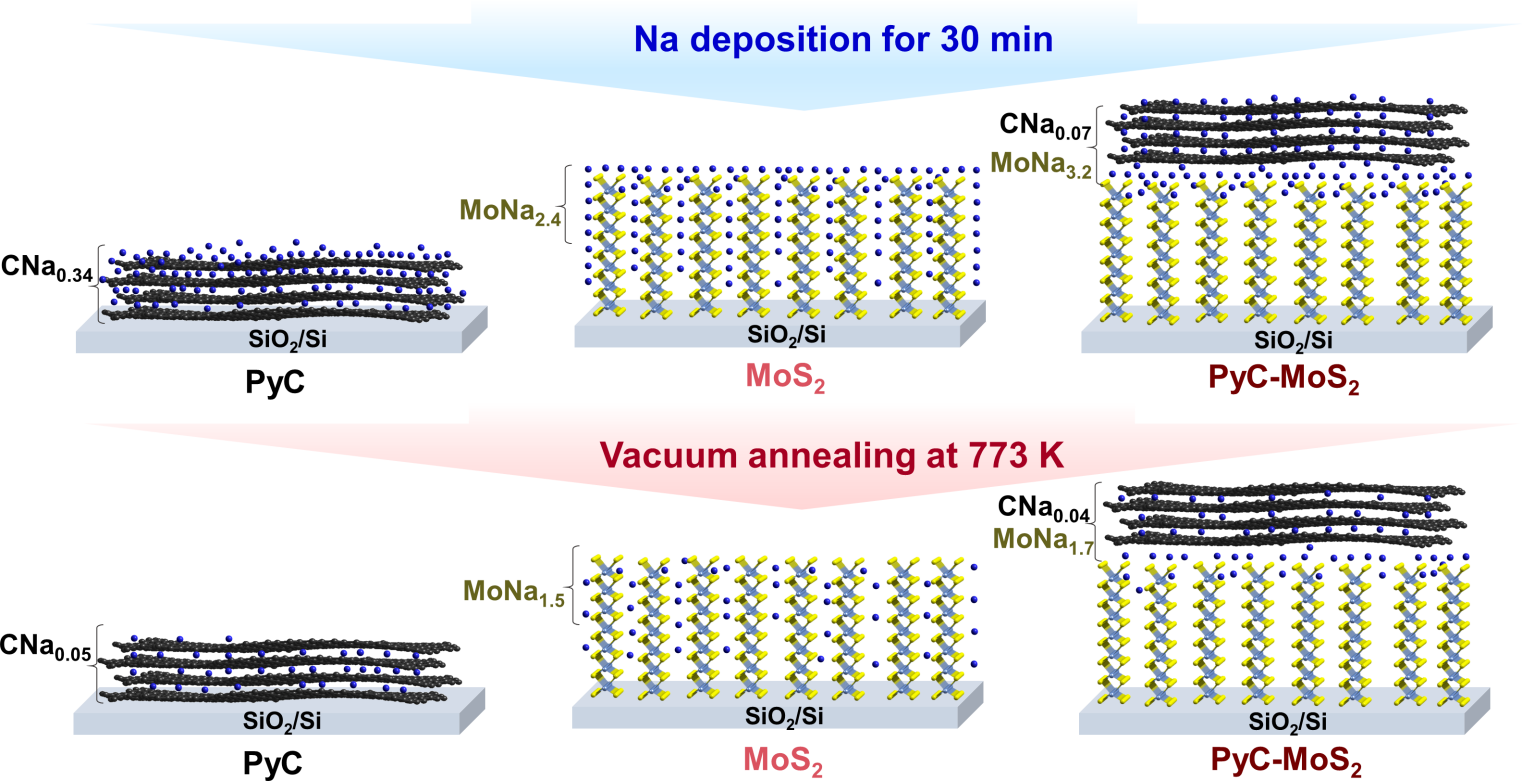
Scheme of the sodium deposition on PyC, MoS_2_, and PyC-MoS_2_ films and subsequent vacuum annealing.

Figure 8 schematically illustrates the difference in the sodium adsorption and desorption on the samples under study. Sodium deposition for 30 min on the PyC film results in a high Na/C ratio of 0.34 at a depth of 3 nm. Sodium is not only adsorbed on the film surface but also accumulates in the film volume, most likely between the graphitic layers. According to the XPS C 1s spectra, an electron density transfer from sodium to carbon occurs. Vacuum annealing of the sodiated PyC film at 773 K removes most of the sodium. The Na/C ratio in the sample is 0.05.

Deposition of the same amount of sodium on the MoS_2_ film gives a Na/Mo ratio of 2.4. Half of the sodium is located on the film surface. The high Na/Mo ratio can be explained by the easy penetration of sodium into the vertically oriented layers of the MoS_2_ film. The XPS Mo 3d spectra reveal that the intercalation of sodium between the MoS_2_ layers leads to a 2H–1T′ transition and electron charge doping from sodium. The Na/Mo ratio decreases after annealing of the sodiated MoS_2_ film because of the partial removal of sodium, primarily from the film surface. After annealing, the sodiated MoS_2_ film still contains a high concentration of sodium in its bulk, since the Na/Mo ratio is 1.5. The annealing conditions used are insufficient to remove all the sodium from the MoS_2_ film and restore its initial 2H structure.

In the case of the PyC-MoS_2_ hybrid, the top PyC layer traps some of the sodium, so the amount of sodium that penetrates into MoS_2_ and accumulates there is less than for the bare MoS_2_ film. It should be noted that the Na/C ratio in the sodiated PyC-MoS_2_ film is approximately five times smaller than in the sodiated PyC film. Sodium atoms prefer to diffuse through PyC to the more attractive MoS_2_, but some of them are retained in the carbon layers. The horizontally oriented graphitic layers act as a barrier and prevent sodium from penetrating into the underlaying MoS_2_ film. In the PyC-MoS_2_ hybrid, sodium accumulation occurs more on the MoS_2_ surface or at the interface between MoS_2_ and PyC, than in the bulk of MoS_2_. Annealing causes sodium to leave the PyC coating to a lesser extent than in PyC alone, but it is released from the MoS_2_ component more readily than from uncoated MoS_2_. It can be concluded that the graphite layers introduced into the MoS_2_ anode material will play a key role in the diffusion and storage of sodium during the charge–discharge of SIBs.

## Conclusion

Synchrotron XPS tool is invoked to study sodium adsorption/desorption in thin films of graphitic PyC, vertically aligned MoS_2_ layers, and PyC-MoS_2_. The MoS_2_ film with a thickness of about 4 nm was synthesized by sulfurization of a molybdenum layer deposited on a SiO_2_/Si substrate using magnetron sputtering. Raman spectroscopy and SEM revealed the vertical orientation of the MoS_2_ layers relative to the substrate surface. According to XPS data, the surface of the MoS_2_ film is enriched with sulfur even after its annealing at 1073 K in hydrogen. PyC films were synthesized by CVD and transferred onto the surfaces of SiO_2_/Si and MoS_2_. PyC, MoS_2_ film, and PyC-MoS_2_ hybrid were used to deposit equal amounts of sodium via evaporation in UHV. Analysis of XPS data revealed a higher sodium concentration on the PyC-MoS_2_ surface than on the MoS_2_ surface since the PyC top layer and the hybrid interface accumulate sodium. Sodium deeply penetrated into the bare MoS_2_ film, causing a transition from the 2H structure to the 1T´ structure due to the transfer of electron density to MoS_2_. Annealing of sodiated samples at 773 K in ultrahigh vacuum resulted in almost complete removal of sodium from PyC and its retention on the surface and in the bulk of the MoS_2_ film. Comparison of MoS_2_ films with and without the PyC coating showed that sodium is released more poorly from the latter. Our findings help explain the electrochemical properties of hybrid anode materials consisting of MoS_2_ and graphite thin layers in SIBs. The presence of PyC protects the surface of MoS_2_ from excess sodium concentration and, consequently, from the destruction of the original MoS_2_ structure.

## Experimental

The substrates cut from a single-crystal silicon wafer were annealed in air at 1323 K for 16 h to form a 250–300 nm thick surface oxidized layer. The substrates were thoroughly cleaned using hot mineral acids and placed in a magnetron sputtering system (OJSC Vacuum Systems). The substrates were annealed at 573 K for 30 min in a vacuum at a pressure of 2 × 10^−2^ Pa. Immediately after this, molybdenum was sputtered from a Mo target with a purity of 99.9% for 10 s at a magnetron power of 100 W and an argon partial pressure of 5.4 × 10^−1^ Pa. The output pressure in the chamber was controlled by the argon flow.

The MoS_2_ films were synthesized by sulfurization of molybdenum layers deposited on SiO_2_/Si substrates in a two-zone quartz reactor. The substrate was placed in the high-temperature zone and annealed there at 423 K for 30 min in an argon flow of 250 sccm. Then, this zone was heated to 873 K. 200 mg of sulfur powder (99.9% purity) were placed in a quartz crucible in the low-temperature reactor zone heated to 473 K. A flow of 24 sccm argon was passed through both reactor zones for 30 min at atmospheric pressure. After this time, sulfurization of the Mo layer was complete. Both zones were cooled to room temperature in a flow of 250 sccm argon. To remove polysulfide impurities and form a more crystallized structure, the MoS_2_ film was annealed in H_2_ atmosphere at 1073 K for 10 min.

PyC films were grown on copper foil at 1273 K for 20 min using low-pressure CVD of methane mixed with hydrogen. The CH_4_ pressure was 800 Pa, and the H_2_ pressure was 2000 Pa. The resulting sample was placed in an aqueous solution of iron chloride (30 wt %) for 2 h to dissolve the copper foil. The remaining free PyC film was washed twice in dilute HCl (10 wt %) and then in deionized water until neutral pH was reached. The floating PyC film was trapped either on bare or MoS_2_-covered SiO_2_/Si substrates and then dried under ambient conditions.

Morphology of sample surfaces was examined by SEM with a CIQTEK SEM5000 (CIQTEK Ltd., Hefei, Anhui, PRC) microscope at an accelerating voltage of 15 kV. The cross section of MoS_2_ film was prepared using a gallium-ion column FIB system and a two-stage protective cap deposition. Initially, a Pt layer of 21 nm was electrodeposited at 5 keV and 1 nA. After that, a thick Pt cap layer was ion-plated at 30 keV and 250 pA. Then, the FIB was operated at an ion accelerating voltage of 30 keV and ion current of 20 nA to cut the sample. Finally, the section was finely polished at an ion current of 250 pA to obtain a smooth surface. The image was acquired using a TESCAN AMBER (TESCAN Ltd., Brno, Czech Republic) microscope at an accelerating voltage of 5 kV in secondary electron mode.

Raman spectra were recorded using a LabRAM HR Evolution spectrometer (Horiba, Kyoto, Japan) using an Ar^+^ laser at a wavelength of 514 nm.

XPS and NEXAFS experiments and sodium deposition were carried out at the RGL-PES end-station of the Russian–German dipole beamline (RGBL dipole) of the Berliner Elektronenspeicherring für Synchrotronstrahlung (BESSY II) operated by Helmholtz-Zentrum Berlin für Materialien und Energie (Berlin, Germany) [56]. Three samples, namely, MoS_2_, PyC, and PyC-MoS_2_ films on SiO_2_/Si substrates were fixed to a holder and placed into UHV (10^−7^ Pa) at the end-station and annealed at 673 K for 10 min to remove the contaminations. NEXAFS C K-edge spectra were acquired by measuring leakage current in total electron yield mode. The experimental data were normalized to the ring current and a photon flux measured using a clean gold crystal.

The XPS spectra were measured at synchrotron radiation of 830 and 470 eV. After the XPS and NEXAFS measurements were completed, the samples were simultaneously exposed to Na vapor from a well-outgassed sodium source (SAES Getters) for 10 min and then again for 20 min (30 min in total) at a current of 7.5 A. XPS measurements of the sodiated samples were performed immediately after each step of Na deposition. To desorb sodium, the samples, after a total of 30 min of Na deposition, were annealed at 773 K for 30 min in UHV. After the annealing procedure, XPS spectra were recorded again. The samples after each step of Na deposition and annealing did not come into contact with air, their transfer between the analytical and preparation chambers was carried out without breaking the vacuum. The energy scale was calibrated using the binding energy of the Au 4f_7/2_ component at 84.0 eV measured from a clean gold foil. The surface concentration of the elements was determined from the XPS survey spectra taking into account the photoelectron cross sections. Shirley background subtraction was used in analysis of fine lines. For the Mo 3d, S 2p, and Na 2s spectra, curve fitting was performed using a Gaussian (40%)/Lorentzian (60%) product function. For the C 1s spectra, the main peak at ≈284.4 eV was fitted using a Lorentzian asymmetric line shape with tail damping, convoluted with a Gaussian function, which closely approximates a Gaussian/Lorentzian product function. Energy position, full width at half maximum, and area for fitted components of the XPS spectra of initial samples are collected in Table S1, Supporting Information File 1.

## Supporting Information

File 1 EDX spectroscopy study of Pt layers protecting MoS_2_ surface, XPS survey spectra of the studied samples, and XPS Na 2s spectra of the sodiated samples.

## Data Availability

All data that supports the findings of this study is available in the published article and/or the supporting information of this article.
