# Supplementary material for: Synchrotron X-ray photoelectron spectroscopy study of sodium adsorption on vertically arranged MoS2 layers coated with pyrolytic carbon
Source: Beilstein J Nanotechnol. 2025 Jun 10;16:847–59. doi: 10.3762/bjnano.16.64 (PMC12207254; doi:10.3762/bjnano.16.64)
Supplement: File 1 — EDX spectroscopy study of Pt layers protecting MoS2 surface, XPS survey spectra of the studied samples, and XPS Na 2s spectra of the sodiated samples. [file Beilstein_J_Nanotechnol-16-847-s001.pdf]

BEILSTEIN JOURNAL OF NANOTECHNOLOGY

Supporting Information

for

Synchrotron X-ray photoelectron spectroscopy study of sodium adsorption on vertically arranged MoS_2 layers coated with pyrolytic carbon

Alexander V. Okotrub, Anastasiya D. Fedorenko, Anna A. Makarova, Veronica S. Sulyaeva, Yuliya V. Fedoseeva and Lyubov G. Bulusheva

Beilstein J. Nanotechnol. **2025,** *16,* 847–859. doi:10.3762/bjnano.16.64

EDX spectroscopy study of Pt layers protecting MoS_2 surface, XPS survey spectra of the studied samples, and XPS Na 2s spectra of the sodiated samples

License and Terms: This is a supporting information file under the terms of the Creative Commons Attribution License (https://creativecommons.org/licenses/by/4.0). Please note that the reuse, redistribution and reproduction in particular requires that the author(s) and source are credited and that individual graphics may be subject to special legal provisions.
The license is subject to the *Beilstein Journal of Nanotechnology* terms and conditions: (https://www.beilstein-journals.org/bjnano/terms)

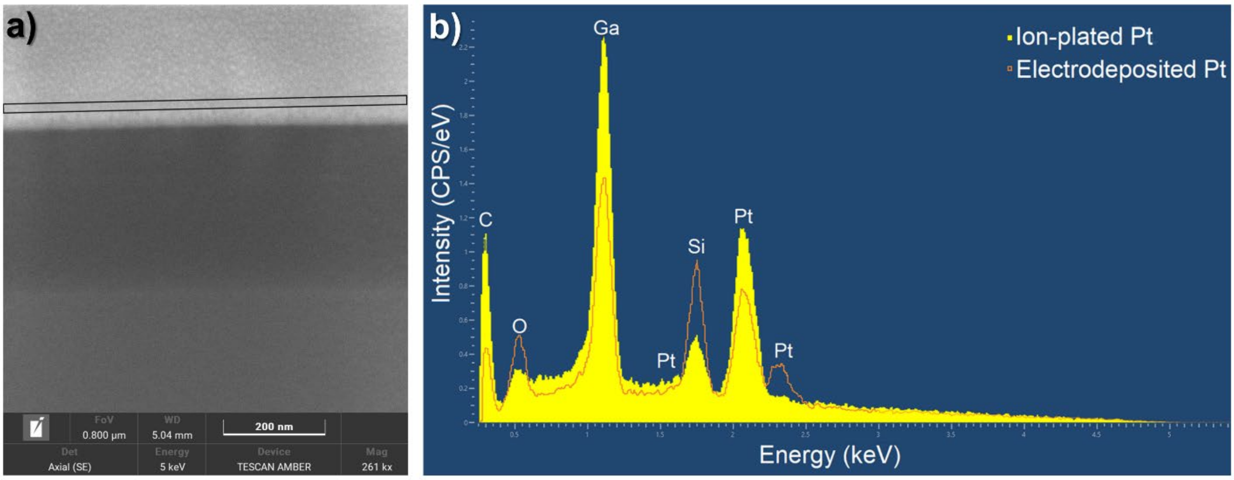

Figure S1: (a) SEM image of the cross-sectional view of the MoS_2 film obtained using a Mo layer sputtered for 90 s. Two horizontal lines show the area for recording EDX spectra. (b) EDX spectra of the electrodeposited Pt layer (orange) and ion-plated Pt layer (yellow).

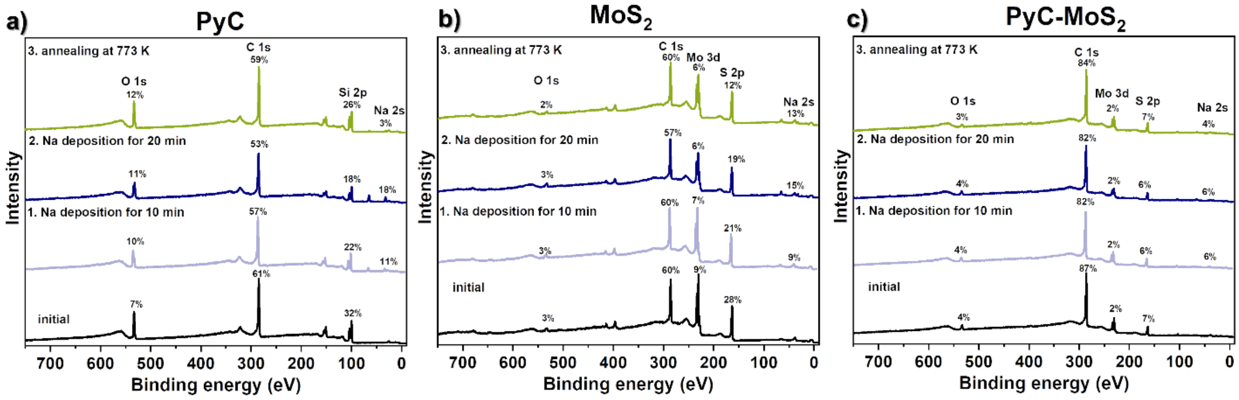

Figure S2: XPS survey spectra of (a) PyC, (b) MoS_2, and (c) PyC-MoS_2 films measured at 830 eV before and after each stage of the sample processing.

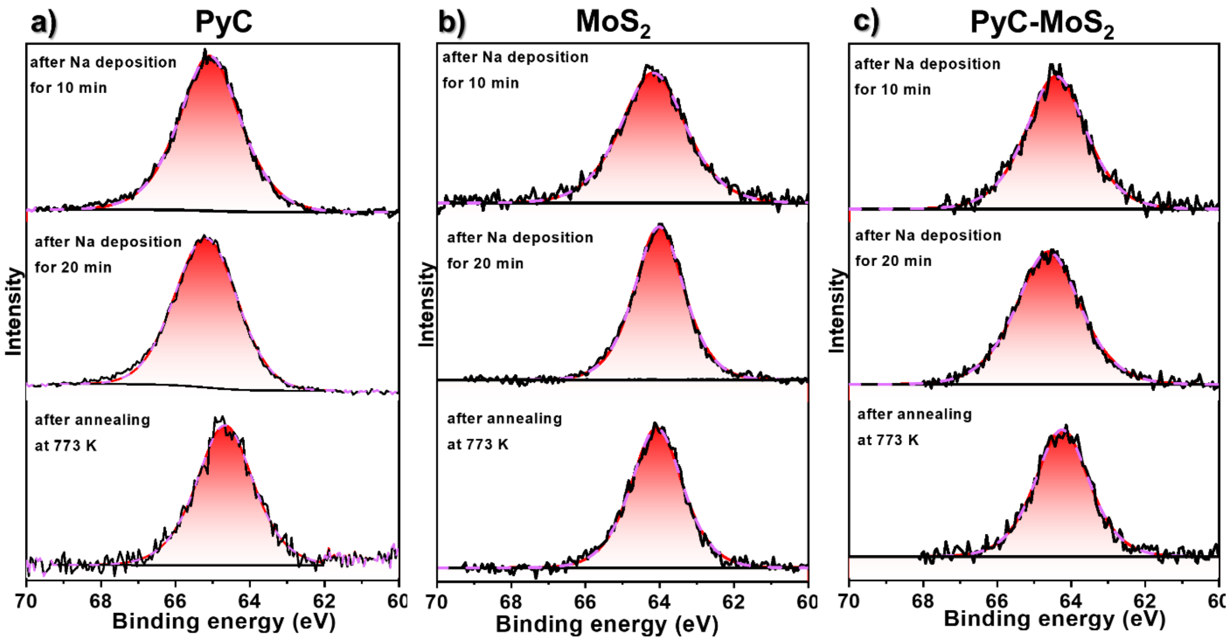

Figure S3: XPS Na 2s spectra measured for (a) PyC, (b) MoS_2, (c) and PyC-MoS_2 samples after sodium deposition and subsequent annealing in UHV.

Table S1: The XPS spectra fitting parameters for initial PyC, MoS_2, and PyC-MoS_2 samples.

Spectrum	Peak assignment	Position, eV	FWHM, eV	Line Shape	Raw Area, CPS
**PyC**					
C 1s	C sp^2	284.4	1.11	LF(0.5,0.9,100,300)	28603
	C-O	286.4	1.68	GL(60)	608
	π* sat.	290.4	2.42	GL(60)	739
**MoS_2**					
Mo 3d	2H-MoS_2 $3d_{5/2}$	228.9	0.87	GL(60)	17696
	2H-MoS_2 $3d_{3/2}$	232.0	1.06	GL(60)	11797
	Mo^{5+} $3d_{5/2}$	230.2	1.24	GL(60)	1456
	Mo^{5+} $3d_{3/2}$	233.4	1.24	GL(60)	971
	Mo^{6+} $3d_{5/2}$	231.7	1.24	GL(60)	972
	Mo^{6+} $3d_{3/2}$	234.9	1.24	GL(60)	648
S 2p	metal rich S $2p_{3/2}$	160.5	1.08	GL(60)	514
	metal rich S $2p_{1/2}$	161.7	1.08	GL(60)	257
	2H-MoS_2 $2p_{3/2}$	161.7	0.83	GL(60)	9813
	2H-MoS_2 $2p_{1/2}$	162.9	0.83	GL(60)	4906
	S_2^{2-} and polysulfide groups $2p_{3/2}$	163.4	1.24	GL(60)	1502
	S_2^{2-} and polysulfide groups $2p_{1/2}$	164.6	1.24	GL(60)	751
**PyC-MoS_2**					
C 1s	C sp^2	284.3	1.17	LF(0.55,0.9,100,300)	37926
	C-O	286.4	1.51	GL(60)	264
	π* sat.	290.0	2.48	GL(60)	796
Mo 3d	2H-MoS_2 $3d_{5/2}$	228.9	0.85	GL(60)	5770
	2H-MoS_2 $3d_{3/2}$	232.1	1.07	GL(60)	3847
	Mo^{5+} $3d_{5/2}$	230.3	1.25	GL(60)	409
	Mo^{5+} $3d_{3/2}$	233.5	1.25	GL(60)	273
	Mo^{6+} $3d_{5/2}$	231.9	1.25	GL(60)	334
	Mo^{6+} $3d_{3/2}$	235.0	1.25	GL(60)	223
S 2p	metal rich S $2p_{3/2}$	160.6	1.05	GL(60)	201
	metal rich S $2p_{1/2}$	161.8	1.05	GL(60)	100
	2H-MoS_2 $2p_{3/2}$	161.8	0.83	GL(60)	3327
	2H-MoS_2 $2p_{1/2}$	163.0	0.83	GL(60)	1664
	S_2^{2-} and polysulfide groups $2p_{3/2}$	160.6	1.05	GL(60)	201
	S_2^{2-} and polysulfide groups $2p_{1/2}$	161.8	1.05	GL(60)	100
